# Raedwoon: A leadership development program for direct care nurses in Saudi Arabia

**DOI:** 10.1097/nmg.0000000000000146

**Published:** 2024-07-02

**Authors:** Nashi Masnad Alreshidi, Afaf Mufadhi Alrimali, Wadida Darwish Alshammari, Kristine Angeles Gonzales, Salwa Thamer Alrashidi, Fe Baltazar Gaspar, Amal Msaid Alrashidi

**Affiliations:** At the Hail Health Cluster, Hail, Saudi Arabia, **Nashi Masnad Alreshidi** is Executive Vice President of Nursing; **Afaf Mufadhi Alrimali** is Head of the Research and Development Department, Nursing Executive Administration; **Wadida Darwish Alshammari** is Head of the Academic Affairs and Training Department, Nursing Executive Administration; **Kristine Angeles Gonzales** is Academic Affairs and Training Department Supervisor, Nursing Executive Administration; **Salwa Thamer Alrashidi** is Nursing Services supervisor, Nursing Executive Administration; **Fe Baltazar Gaspar** is Academic Affairs and Training Department Supervisor, Nursing Executive Administration. **Amal Msaid Alrashidi** is Continuing Nursing Education Supervisor at Hail General Hospital in Hail, Saudi Arabia.

## Abstract

This study assessed the Raedwoon program, a 25-day nursing leadership course, using a quasi-experimental design. It found overall improvements in leadership and nursing competencies, except in the “Inspire a Shared Vision” practice and documentation skills.

**Figure:**
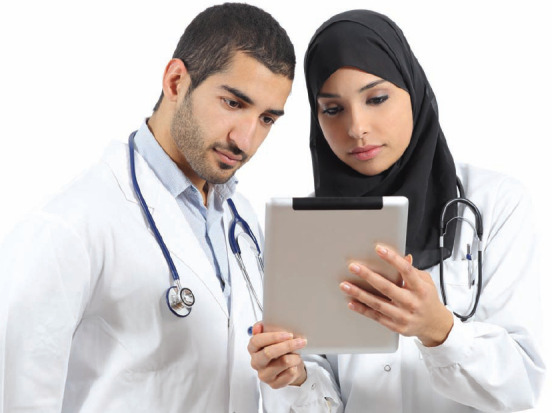
No caption available.

Nurses are widely recognized as the cornerstone of healthcare ecosystems, tasked with overcoming the complex challenges of their profession under the guidance of skilled leaders. Research has consistently shown that effective leadership is not only foundational to organizational success, but also enhances nurse satisfaction and retention, in addition to having a profound impact on healthcare delivery and patient outcomes.^1^-^4^ However, the healthcare sector faces significant challenges due to a shortage of nurse leaders and an increasing trend of nurse managers leaving their positions.^5^ In addition, the proportion of nurses expressing a lack of interest in leadership roles and a perceived unpreparedness to take on such positions is accelerating notably.^6^ Variables linked to diminished interest in leadership roles among nurses included a lack of readiness for leadership and insufficient preparation.^7^ These issues primarily affect the retention and recruitment of qualified nurses who can assume and excel in these vital leadership roles.^3^,^8^

This situation has prompted the creation of targeted educational programs aimed at enhancing nurses' leadership abilities. Such programs can cultivate nurses' leadership qualities and pave the way for career growth, empowering nurses to assume crucial leadership positions and make policy decisions that influence the course of healthcare delivery.^1^,^9^ The lack of nursing leadership and the shortage of reliable workforce data in recent years have stunted the growth of the nursing field in Saudia Arabia, causing it to lag behind international nursing advancements.^10^ This gap highlights the urgent need for leadership education research, which seeks to address these shortcomings by nurturing leadership skills within the Saudi Arabian nursing community and promoting alignment with global nursing standards and practices.

## The Raedwoon leadership educational program

The Raedwoon Program is specifically developed to align with the unique requirements of the local healthcare system. Initiated by the Nursing Executive Administration within Hail Health Cluster, which is the entity responsible for providing healthcare services in Hail region, Saudi Arabia, its primary objective is to bolster nursing services by identifying and developing leadership potential. The program derives its name from the Arabic term for “leaders,” reflecting its focus on nurturing a cadre of nurse leaders capable of adeptly managing the demands of contemporary healthcare. Educational initiatives rooted in established theoretical models and tailored to the specific contexts of their organizations are reported to be effective in developing nursing leadership.^1^ Therefore, the Raedwoon Program integrates the “Five Practices of Exemplary Leadership” conceptualized by James Kouzes and Barry Posner, adapting its principles to the nursing context.^11^ The program is focused on cultivating the knowledge, skills, and essential competencies of emerging leaders, thereby fostering a reservoir of skilled individuals for nursing leadership positions. This aligns with the Hail Health Cluster's strategic objective to “develop leadership and talent,” as part of the strategic theme to “build the foundations and capabilities of the future.”

Spanning 25 days, the Raedwoon Program offers a comprehensive and multifaceted curriculum designed to enhance nursing leadership, including interactive group exercises such as case studies, role-playing scenarios, and simulation exercises that foster collaborative learning and problem-solving skills. The program covers a broad spectrum of topics including advanced nursing practices, healthcare policy, leadership ethics, communication strategies, accreditation standards, and change management in healthcare settings. A strong emphasis is placed on developing skills such as emotional intelligence, conflict resolution, team building, and motivation. Integral to the curriculum is the development of core competencies in nursing, with a particular focus on clinical judgment, patient safety, and evidence-based practice, ensuring that participants are not only leaders but also highly competent practitioners.

Key to the program is the hands-on education in both administrative and clinical settings. Administrative education encompasses healthcare administration, resource management, and team leadership, whereas clinical education focuses on patient-care management, clinical decision-making, and emergency response strategies. A distinctive feature is the practical observation phase under the mentorship of seasoned nurse leaders such as the director of nursing, nursing supervisor, nursing quality management supervisor, continuing nursing education supervisor, and head nurses (nurse managers). This phase provides shadowing experiences and personalized coaching sessions, offering insights into various nursing roles and leadership styles. Participants are also encouraged to undertake a capstone project to apply their learned knowledge and skills by identifying and addressing real-world challenges in their work environments.

The Raedwoon Program incorporates Kouzes and Posner's five practices of exemplary leadership in various practical and engaging ways:

1. **Model the Way:** Participants engage in self-reflection exercises and develop personal leadership philosophy statements, which help them identify and solidify their values and beliefs. These activities are complemented by shadowing experienced nurse leaders during the practical observation phase, during which they witness firsthand how values and principles are embodied in daily leadership practices.
2. **Inspire a Shared Vision:** Through workshops focused on communication, participants learn to develop and articulate a compelling vision for their teams. They practice crafting and delivering inspirational messages, using case studies that involve creating a vision for change or improvement in healthcare settings.
3. **Challenge the Process:** The curriculum includes problem-solving workshops in which participants analyze case studies involving complex healthcare scenarios. They're encouraged to think critically and propose innovative solutions, thereby challenging existing norms and practices in healthcare.
4. **Enable Others to Act:** Group exercises and role-playing scenarios are used to simulate team management situations. Participants practice delegation, empowering team members, and fostering collaboration. They also receive education in conflict resolution, learning to navigate and resolve interpersonal challenges within a team.
5. **Encourage the Heart:** The program incorporates sessions on recognizing and valuing the contributions of team members. Participants engage in activities that focus on building team morale and showing appreciation, such as learning how to give effective, positive feedback and celebrating small wins in team projects.

Each element of the program is crafted to provide practical, real-world applications, ensuring that graduates are well-equipped to lead effectively and empathetically in the nursing field.

## Purpose of the study

The scarcity of research on leadership program development within Saudi Arabia underscores the urgent need for scholarly investigation into this field. This article presents a study that assesses the impact of the Raedwoon Program on the enhancement of leadership skills and professional nursing practices. This study goes beyond merely identifying the presence of leadership skills, delving into the evaluation of nursing competencies to ensure that nurses not only have the skills to lead but also adhere to professional standards and guidelines, are prepared to manage effective teams, and contribute positively to patient outcomes. By examining the experiences of 29 RNs who participated in the program, this study aims to expand the body of knowledge on nursing leadership development, and by doing so, it directly responds to the highlighted gap in the literature.

## Methods

### Study design

A quasi-experimental design was employed to evaluate the effectiveness of the Raedwoon Program, with a single group undergoing pre- and posttest. The institutional review board at Hail Region approved the study: approval number (2023-57).

### Sample and setting

The investigators recruited participants for the Raedwoon Program from all 16 public hospitals within Hail Health Cluster. They determined the sample size of 29 nurses based on factors such as the expected effect size, standard deviation, and a standard alpha level of 0.05. These 29 nurses represented the entire Raedwoon Program class and 100% of eligible nurses within the recruitment period. All participants completed both the pre- and posttests, enabling a consistent and comprehensive assessment of the program's impact on their leadership development. To be eligible for the program, candidates were required to hold a bachelor's of science in nursing (BSN) degree and have a minimum of 3 years of experience as staff nurses. Candidates also needed to demonstrate leadership potential, based on their performance appraisals. Those without a formal BSN qualification or with less than 3 years of postqualification experience were excluded. Despite not being in formal leadership roles, many participants had informal leadership experience, such as leading teams or initiatives, providing a foundation for further development through the program. Recruitment was conducted via hospital communications, with applicants undergoing a thorough selection process involving standardized interviews by a diverse panel of experts to ensure unbiased evaluations. The focus on diversity and inclusion aimed to attract a broad range of participants, with decisions made based on their qualifications, experience, and fit with the program's objectives.

### Data collection

Data were collected between August and September 2023 using a web-based survey. Informed consent was obtained from all participants. The participants were provided with detailed information about the study's aims; the voluntary nature of their participation, potential risks, and benefits; the confidentiality and anonymity of the data collected; and the estimated time required for survey completion.

The investigators assessed the demographic variables of the participants to understand the diversity of the sample and control for potential confounding variables. Demographic variables included gender, age group, education level, years of experience, professional title, and unit of work. To determine the program's effectiveness, the investigators conducted a baseline assessment using two key tools: the Leadership Practices Inventory (LPI) and the Nurse Professional Competence (NPC) Scale. These tools were chosen to measure changes in leadership practices and nursing competence, respectively. Following the completion of the Raedwoon leadership program, the same assessment was administered to the same group of participants to evaluate any changes or improvements in their leadership practices and overall nursing competence.

### Instrument

Created by Kouzes and Posner in the early 1980s, the LPI evaluates leadership competencies through five key practices: Modeling the Way, Inspiring a Shared Vision, Challenging the Process, Enabling Others to Act, and Encouraging the Heart. It uses a 10-point scale to measure the occurrence of 30 distinct leadership actions, with each of the five practices corresponding to six specific behaviors. Participants self-assess the frequency of these behaviors, with scores ranging from 1 (almost never) to 10 (almost always). The aggregate score for each practice is derived by totaling the scores of the six associated behaviors, giving a possible range of 6 to 60 for each practice. The LPI demonstrates strong reliability, with Cronbach alpha values exceeding 0.70 for each of the five leadership practices categories.^12^

To evaluate the nurses' competence, the short version of the NPC Scale was utilized. Originating in Sweden, the comprehensive NPC Scale includes 88 questions distributed across eight competence areas (CAs), as described by Nilsson and colleagues.^13^ The NPC Scale Short Form (NPC-SF) was created in 2018, and it consists of 35 items measuring six CAs using a 4-point Likert-type scale, ranging from 1 (to a very low degree) to 4 (to a very high degree).^14^ Nurses are also given a “can't decide” response option. The six areas of competence assessed by the scale are: Nursing Care; Value-Based Nursing Care; Medical and Technical Care; Care Pedagogy; Documentation and Administration of Nursing Care; and Development, Leadership, and Organization of Nursing Care. The reliability of the NPC-SF is deemed acceptable, with Cronbach alpha values ranging between 0.71 and 0.86.^14^,^15^

### Data analysis

The collected data were compiled and transferred into a Microsoft Excel spreadsheet for analysis. Then they were cleaned, recoded, and analyzed using IBM SPSS version 29.0. Descriptive statistics were produced. Continuous variables were expressed as means ± standard deviation. Independent samples *t* tests and analysis of variance (ANOVA) were used to compare the means of the continuous variables against those of the nominal variables. Paired sample *t* tests were employed to analyze the discrepancies in the data between the pre- and posttest results. Data normality was assessed, and the assumptions were satisfied. All analyses were calculated with a 95% confidence interval and considered statistically significant at *P* < .05.

## Results

A total of 29 responses were analyzed. Participants' personal and professional characteristics varied (see Table 1). Most participants were female (65.5%), and the predominant age group was 20 to 29 (62.1%). With respect to education, a significant majority held a bachelor's degree (93.1%). Experience levels were mainly between 3 and 5 years (51.7%), and staff nurses comprised the largest group (48.3%). The most common work setting was non-ICU (37.9%). Table 2 shows the mean, median, and standard deviation of the five leadership practices generated from the LPI questionnaire and the six constructs from the NPC Scale.

**Table 1:** Personal and work-related characteristics of the participants (N = 29)

Characteristic	Frequency (%)
**Sex**	
Female	19 (65.5)
Male	10 (34.5)
**Age (years)**	
20-29	18 (62.1)
30-39	10 (34.5)
40-49	1 (3.4)
**Education**	
Bachelor's degree	27 (93.1)
Master's degree	2 (6.9)
**Experience**	
3 to 5 years	15 (51.7)
5 to 10 years	11 (37.9)
10 to 15 years	3 (10.3)
**Job title**	
Staff nurse	14 (48.3)
Head/charge nurse	11 (37.9)
Other	4 (13.8)
**Unit**	
Non-ICU	11 (37.9)
ICU	8 (27.6)
Other	10 (34.5)

**Table 2:** Distribution of the mean values, median values, and standard deviation (SD) of the five LPI items and the six NPC Scale items

Practice	Mean	Median	SD
*LPI*			
Model the Way	54.4	57.0	6.7
Inspire a Shared Vision	23.0	24.0	1.9
Challenge the Process	54.8	57.0	5.9
Enable Others to Act	55.4	58.0	6.1
Encourage the Heart	55.1	58.0	6.2
*NPC Scale*			
Nursing Care	73.8	76.0	7.6
Value-Based Nursing Care	76.3	80.0	6.8
Medical and Technical Care	63.6	66.6	6.1
Care Pedagogics	76.3	80.0	7.1
Documentation and Administration of Nursing Care	48.0	80.0	7.09
Development, Leadership, and Organization of Nursing Care	62.7	66.6	7.2

Concerning the LPI, the top leadership practices reported by the respondents, at more than 70%, were Challenge the Process, Model the Way, Encourage the Heart, and Enable Others to Act. However, there was a slight emphasis on the Encourage models of practice (see *Supplementary Table 1*, http://links.lww.com/NMT/A4). The five NPC items receiving the highest scores were in the areas of Value-Based Nursing Care and Care Pedagogics, while the five lowest-scoring items were in Documentation and Administration of Nursing Care (see *Supplementary Table 2*, http://links.lww.com/NMT/A5).

When comparing the demographic characteristics related to the five leadership practices, there were no statistically significant differences based on participants' education, experience, title, or unit of practice. There were, however, differences by gender and age. Females scored higher than males in the Inspire, Challenge, and Encourage practices. Those in the youngest age group scored higher than the others in the Inspire, Enable, and Encourage practices (see Table 3). Related to the NPC Scale, there were also no statistically significant differences based on the participants' education, experience, title, or unit of practice. Females scored higher in every construct of the NPC Scale, except Nursing Care, which wasn't a statistically significantly difference. The scores in Value-Based Nursing Care also differed by age, with scores descending with increasing age. Nursing Care was also statistically different based on the unit of practice, with those working in the ICU having the highest scores (see Table 3).

**Table 3:** Demographic characteristics by LPI and NPC scores

	LPI					NPC Scale					
Characteristic	Model the Way	Inspire a Shared Vision	Challenge the Process	Enable Others to Act	Encourage the Heart	Nursing Care	Value-Based Nursing Care	Medical and Technical Care	Care Pedagogics	Documentation and Administration of Nursing Care	Development, Leadership, and Organization of Nursing Care
Gender											
Male	51.80	21.70∗	51.60∗	52.90	52.00∗	71.20	72.40∗	59.70∗	71.20∗	45.16∗	56.92∗∗
Female	55.79	23.63∗	56.47∗	56.68	56.79∗	75.16	78.32∗	65.62∗	78.95∗	49.42∗	65.87∗∗
Age Group											
20-29	54.56	23.44∗	55.00∗	56.22	56.06∗	74.89	78.67∗	65.25	78.00	48.87	64.19
30-39	55.40	22.60∗	55.70∗	56.00	55.50∗	72.40	73.20∗	61.09	74.00	46.72	61.09
40-49	42.00	18.00∗	42.00∗	34.00	35.00∗	68.00	64.00∗	58.31	68.00	43.75	52.76
Education											
Bachelor's	54.74	22.93	54.81	55.52	55.19	73.78	76.44	63.55	76.44	47.86	62.48
Master's	50.00	23.50	54.50	53.50	54.50	74.00	74.00	63.86	74.00	49.22	65.25
Experience, y											
2-5	55.13	23.20	55.47	55.93	55.60	73.87	77.60	64.23	77.07	48.13	62.87
5-10	52.82	22.91	53.18	54.00	53.91	74.18	76.00	63.61	76.00	48.30	62.85
10-15	56.67	22.00	57.33	57.67	57.33	72.00	70.67	60.16	73.33	45.83	61.09
Job title											
Staff nurse	52.64	22.50	52.86	52.79	52.93	71.43	74.86	62.87	74.00	47.32	60.89
Head/charge nurse	56.73	23.18	56.91	58.00	57.55	75.27	76.73	63.36	77.82	48.01	63.86
Other	54.25	24.00	55.75	57.25	56.25	78.00	80.00	66.64	80.00	50.00	66.64
Unit											
ICU	56.75	23.75	56.50	57.75	57.38	77.50∗	79.50	66.29	78.00	49.22	64.90
Non-ICU	54.00	23.00	54.82	54.82	55.45	75.27∗	75.64	63.61	76.00	48.30	63.61
Other	53.00	22.30	53.40	54.10	53.00	69.20∗	74.40	61.36	75.20	46.56	59.54

∗ *P* < .05

∗∗ *P* < .001

From the pre- to posttest, there were significant changes in LPI scores among the five leadership practices; *P* < .05. All practice scores increased between 5.31 and 7.41 points, except Inspire a Shared Vision, which decreased by a mean of 26.03 points (see Table 4). A similar pattern was noticed with the pre- versus post-NPC scores, with significant increases ranging from 6.31 to 7.50 points for all scales (see Table 4).

**Table 4:** LPI and NPC scores pretest versus posttest

Practice	Mean difference	SD	*P* value
*LPI*			
Model the Way	7.41	8.30	<.001
Inspire a Shared Vision	-26.03	7.03	<.001
Challenge the Process	6.14	6.86	<.001
Enable Others to Act	5.31	7.74	<.001
Encourage the Heart	3.83	4.94	<.001
*NPC Scale*			
Nursing Care	7.11	11.60	.002
Value-Based Nursing Care	6.62	13.67	.010
Medical and Technical Care	7.52	13.34	.006
Care Pedagogics	7.29	12.78	.003
Documentation and Administration of Nursing Care	6.31	8.11	<.001
Development, Leadership, and Organization of Nursing Care	7.50	11.16	.001

In the follow-up phase of the evaluation, the investigators collected data on the current professional roles of the participants to assess career advancement after program completion. The findings revealed that the professional roles within the cohort were quite diverse, reflecting significant career progression. A noteworthy 24.14% of participants had advanced to the position of charge nurse, while 17.24% had achieved the head nurse role, and 6.90% had been promoted to director of nursing roles. Additionally, participants had attained the positions of supervisor and program deputy director.

## Discussion

The study revealed an upward trend in both LPI and NPC scores from the pretest to the posttest, highlighting the intervention's overall effectiveness. Interestingly, there was a marked decrease in the Inspire a Shared Vision practice on the LPI postprogram. The most prominent leadership practices were Challenge the Process, Model the Way, Encourage the Heart, and Enable Others to Act, with a particular emphasis on Encourage the Heart. In terms of the NPC Scale, the highest scores were recorded in Value-Based Nursing Care and Care Pedagogics, whereas the lowest scores centered around Documentation and Administration of Nursing Care. Demographically, gender and age showed significant variations in both the LPI and NPC Scale, with females outscoring males in several areas and younger respondents displaying a propensity toward certain leadership practices.

The pre/posttest evaluations provide significant insights into the evolution of leadership practices. It's noteworthy that all leadership practices, except Inspire a Shared Vision, witnessed an upswing. This implies that by the end of the program, the participants were potentially more attuned to and capable of executing recognized leadership actions. Contrary to our observations, most past studies have reported improvements across all five LPI subscales.

For instance, Leggat and colleagues observed considerable growth in all LPI subscales among Australian nurses whose training emphasized inquiry-based learning, combining simulations, online modules, and workshops.^16^ In the US, Bhalla and colleagues noted an overall increase in LPI subscale scores among participants of 90-minute seminars based on the Nursing Leadership Institute Competency Model.^17^ However, Spencer and colleagues, whose Association Development and Professional Transformation workshop blended transformational leadership with Kouzes and Posner's 2012 Leadership Challenge framework, reported changes in LPI subscales that weren't statistically significant.^18^ On the other hand, Fitzpatrick and colleagues highlighted that significant enhancements in leadership practices among American nurses were sustained 3 months after the program.^19^ The focus of Fitzpatrick and colleagues' study was on refining nurses' clinical leadership skills, such as personal awareness and change management, although the precise educational framework wasn't specified. Each program, distinct in its structure and emphasis, offers unique perspectives on leadership development among nurses, as evidenced through LPI assessments.

The results derived from the LPI highlight the respondents' strong inclination toward certain leadership practices, specifically Challenge the Process, Model the Way, Encourage the Heart, and Enable Others to Act, with a particular emphasis on the practices of Encourage the Heart. From this, it can be inferred that these nurses foster an environment in which innovation is cherished, setting an example becomes integral, and ensuring continuous motivation becomes a cornerstone of their leadership style. These observations counter studies conducted in Brazil and the US, where participants displayed proficiency in all five leadership tenets.^20^,^21^ However, what stands out in those studies is the pronounced emphasis on the practice of Enabling Others to Act, underscoring the priority that leaders place on empowering team members and reinforcing the universal importance of collaborative work environments.

In this study, the notable decline in Inspire a Shared Vision merits deeper investigation. This drop could stem from the intervention's design or content, which may lack emphasis on this leadership aspect. Alternatively, participants' evolving understanding postintervention might have made them more self-critical about their ability to inspire a shared vision. Their perceptions may have shifted, leading them to deem other leadership practices more vital. External organizational factors could also influence this change. Further qualitative analysis and participant feedback are needed to discern the exact cause.

The pre- to posttest evaluations revealed a significant increase across all NPC Scale items, indicating that the intervention was predominantly effective in enhancing nursing competence. This positive trend aligns with findings from Høegh-Larsen and colleagues who observed similar improvements among nursing students in Norway.^22^ Notably, our results emphasize nurses' self-perceived competence in Value-Based Nursing Care and Care Pedagogics. This underscores the inherent emphasis nurses place on the ethical and educational dimensions of their roles. Several European studies found similar high scores in Value-Based Nursing Care.^22^-^25^ However, in a divergent trend, nurses in Saudi Arabia recorded lower competencies in Care Pedagogics, while both Saudi and European nurses perceived Development, Leadership, and Organization of Nursing Care as areas of lesser proficiency.^15^,^22^,^25^ This could be attributed to the multifaceted nature of those competencies, as they require not only clinical expertise but also organizational skills, leadership abilities, and a deep understanding of healthcare systems.

Meanwhile, our findings pinpoint a potential weak link in the chain: Documentation and Administration of Nursing Care. Despite being pivotal to patient-care continuity and interprofessional communication, this area was perceived as a lesser competency. One potential rationale might be that the growing administrative requirements in healthcare are diverting nurses' attention from their core care duties. Alternatively, it might suggest a need for more comprehensive educational modules on these tasks, perhaps calling for system-level interventions to simplify and streamline administrative processes, thus reducing the perceived burden on the nursing cadre.

The demographic comparisons present interesting insights. The absence of any statistically significant variance across parameters such as education, experience, title, or unit of practice with regards to the LPI and NPC signifies that these factors might not necessarily dictate leadership practices or the perception of professional competencies. Gender and age, however, introduced pronounced differences. For instance, females showed a higher propensity to the Inspire, Challenge, and Encourage practices, which aligns with the literature suggesting that women often adopt transformational leadership styles, characterized by inspiration and motivation.^26^

Moreover, the trend whereby younger respondents scored higher in the Inspire, Enable, and Encourage practices might imply a generational shift in leadership practices, or it could be a reflection of the enthusiasm and adaptability often associated with younger professionals.^27^ From the NPC perspective, females consistently outscored males in almost every construct, again highlighting potential gender-based nuances in nursing professionalism. The declining trend in Value-Based Nursing Care with increasing age is intriguing, potentially suggesting that, as professionals age, their perspectives or priorities shift. Similar findings were reported by Bjuresäter and colleagues.^25^

### Limitations

When interpreting the results of this quasi-experimental study, it's crucial to acknowledge the absence of a control group. This omission leaves the study vulnerable to external factors. Additionally, the study was limited to LPI self-assessments due to resource constraints, acknowledging that a 360-degree assessment may offer a more holistic evaluation of leadership practices. Furthermore, the study didn't incorporate a qualitative assessment, which would have provided deeper insights into underlying trends and participant perspectives.

## Implications for nurse leaders

Nursing leadership stands at the forefront of advancing healthcare quality and fostering innovation. Effective education leadership programs are vital in nurturing these roles, with a stronger emphasis on experiential learning and mentorship. It's important to tailor these programs to address specific competency gaps, such as in documentation and administration, and to account for demographic influences on leadership styles and competencies. Leadership education shouldn't focus only on enhancing existing strengths but also on identifying and improving areas that require more attention, such as visionary leadership skills. Finally, establishing continuous professional development pathways is paramount to ensure the longevity and adaptability of leadership competencies in an evolving healthcare landscape.

## Sustained leadership excellence

The comprehensive evaluation of leadership practices, underscored by the pre- to posttest evaluations, illustrates the program's efficacy. Participants have assumed a spectrum of leadership roles postprogram—evidence of the program's impact on nurturing pivotal leadership skills and fostering professional growth. This outcome affirms the intervention's effectiveness, but other findings highlight specific areas requiring further scrutiny. Notably, although most nurse participants have enhanced their leadership practices, the domain of Inspiring a Shared Vision has emerged as an exception, signaling a need for more targeted analysis to refine the program's future versions.

Furthermore, we've identified a competence gap in documentation practices, potentially indicative of wider systemic challenges or educational shortcomings that merit strategic interventions. The data also reveal the significant influence of demographic variables such as gender and age on leadership styles and competencies. These demographic nuances are more pronounced in certain groups and have been shown to shape leadership perceptions.

It's essential to recognize that although continuous professional development and targeted training can meaningfully transform leadership perceptions, a comprehensive understanding of the root causes and broader implications of these changes is crucial. Such an understanding will guide the development of nuanced interventions that not only address immediate skill gaps but also foster an environment conducive to sustained leadership excellence in nursing.
